# Evidence for Anomalous Network Connectivity during Working Memory Encoding in Schizophrenia: An ICA Based Analysis

**DOI:** 10.1371/journal.pone.0007911

**Published:** 2009-11-19

**Authors:** Shashwath A. Meda, Michael C. Stevens, Bradley S. Folley, Vince D. Calhoun, Godfrey D. Pearlson

**Affiliations:** 1 Olin Neuropsychiatry Research Center, Institute of Living at Hartford Hospital, Hartford, Connecticut, United States of America; 2 Department of Psychiatry, Yale University School of Medicine, New Haven, Connecticut, United States of America; 3 Department of Psychiatry, Johns Hopkins University, Baltimore, Maryland, United States of America; 4 The MIND Institute, Albuquerque, New Mexico, United States of America; 5 Vanderbilt University, Kennedy Center and Department of Psychiatry, Nashville, Tennessee, United States of America; Ecole Polytechnique Federale de Lausanne, Switzerland

## Abstract

**Background:**

Numerous neuroimaging studies report abnormal regional brain activity during working memory performance in schizophrenia, but few have examined brain network integration as determined by “functional connectivity” analyses.

**Methodology/Principal Findings:**

We used independent component analysis (ICA) to identify and characterize dysfunctional spatiotemporal networks in schizophrenia engaged during the different stages (encoding and recognition) of a Sternberg working memory fMRI paradigm. 37 chronic schizophrenia and 54 healthy age/gender-matched participants performed a modified Sternberg Item Recognition fMRI task. Time series images preprocessed with SPM2 were analyzed using ICA. Schizophrenia patients showed relatively less engagement of several distinct “normal” encoding-related working memory networks compared to controls. These encoding networks comprised 1) left posterior parietal-left dorsal/ventrolateral prefrontal cortex, cingulate, basal ganglia, 2) right posterior parietal, right dorsolateral prefrontal cortex and 3) default mode network. In addition, the left fronto-parietal network demonstrated a load-dependent functional response during encoding. Network engagement that differed between groups during recognition comprised the posterior cingulate, cuneus and hippocampus/parahippocampus. As expected, working memory task accuracy differed between groups (*p*<0.0001) and was associated with degree of network engagement. Functional connectivity within all three encoding-associated functional networks correlated significantly with task accuracy, which further underscores the relevance of abnormal network integration to well-described schizophrenia working memory impairment. No network was significantly associated with task accuracy during the recognition phase.

**Conclusions/Significance:**

This study extends the results of numerous previous schizophrenia studies that identified isolated dysfunctional brain regions by providing evidence of disrupted schizophrenia functional connectivity using ICA within widely-distributed neural networks engaged for working memory cognition.

## Introduction

Working memory refers to the temporary retention of information to solve problems or guide behavior. Neuroimaging studies [1], direct intracellular recordings [2], and lesion studies support neurobiological models that [1], [3] emphasize the importance of activity in prefrontal cortex and parietal brain regions [4] within a complex architecture of different anatomical regions associated with temporally distinct phases of working memory (e.g., encoding, rehearsal, and retrieval). Working memory dysfunction in schizophrenia is a prominent neuropsychological deficit and is considered to be a promising endophenotypic marker to better understand the pathology and risk for the disorder [5], [6], [7]. People with schizophrenia reliably show deficits on a variety of working memory tasks [8], [9]. Our previous results [10] suggest that working memory deficits are prominent during encoding, especially in the dorso- and ventrolateral PFC, posterior parietal regions, cingulate and basal ganglia [11], [12], [13]. This is consistent with previous studies showing reduced cerebral perfusion during encoding of episodic memory in the above mentioned areas [14], [15]. Most of the brain regions involved with memory encoding also have been observed to display abnormal activity in schizophrenia during working memory maintenance or delay periods [1], [16]. Similarly, several brain regions including the dorsolateral PFC, visual association, cingulate and hippocampus that have been shown to be anomalous in schizophrenia during recognition/retrieval often overlap with stimulus encoding [15], [17]. This might suggest that despite conceptual differences between encoding and recognition, the same networks/regions that behave abnormally during encoding in schizophrenia probably are also affected during recognition.

Because successful working memory involves the recruitment of multiple task-specific regions to mediate cognitive demands, it is plausible that working memory abnormalities in schizophrenia are associated with improper functional integration between these various task related networks, rather than by failure of a single region [“disconnection hypothesis”; [18], [19]]. Such functional disconnection abnormalities are best demonstrated using specialized analytic approaches such as independent component analysis (ICA) [20] that reveal profiles of integrated neural circuitry instead of simple identification of brain activity seen in conventional fMRI analyses. The patterns of functional integration underlying working memory have become fairly well-characterized in several fMRI functional connectivity studies that found that brain regions identified by conventional fMRI are functionally integrated during working memory performance [7], [21], [22], [23], [24]. Gruber et al. [7], using a psychopysiological interaction (PPI) approach, found a neural circuit comprised of ventrolateral prefrontal cortex (i.e., Broca's Area) and ventral premotor cortex that was engaged during the encoding phase of a verbal working memory task and a posterior parietal-prefrontal network that was recruited during information maintenance. Woodward et al. [23] used a novel constrained-Principal Component Analysis approach to identify how functional networks were differentially engaged by temporally distinct phases of a working memory task. They reported that a bilateral dorsolateral prefrontal (DLPFC)-bilateral superior parietal-anterior cingulate-occipital circuit engaged during encoding was load- dependent (increased condition-specific regression weights with increased loads). In addition, a predominantly left-hemisphere lateralized circuit of prefrontal-posterior -dorsal cingulate regions was engaged during active manipulation of information. Babiloni and colleagues [21] used an EEG coherence analysis to determine working memory network relationships and found increased fronto-parietal connection during short term memory processes compared to baseline condition. Collectively, these studies consistently implicate a functionally integrated circuit consisting of cingulate-dorso/ventro-lateral PFC-posterior parietal-occipital regions to be actively engaged during encoding [7], [23], [24], [25]. Only one study so far examined functional connectivity during working memory recognition/retrieval, which found load-dependent functional integration of an inferior parietal- anterior cingulate-middle occipital – pre-frontal cortex circuit [25].

There have been a handful of functional connectivity studies of schizophrenia [26], [27], [28], but none have specifically examined working memory. Stephan et al [26] showed that olanzapine improved impaired cerebellar-prefrontal-mediodorsal thalamus connectivity in schizophrenia. Other studies found schizophrenia-related disconnection among PFC and other regions. For instance, Das et al [27] examined fear processing in schizophrenia and found a reversal of the normal connectivity patterns between the amygdala, anterior cingulate and the dorsal and ventral divisions of the medial prefrontal cortex. Zhou et al [28] found reduced functional connectivity in first-episode schizophrenia between DLPFC-posterior cingulate-parietal lobe-basal ganglia circuit using passive “resting state” data. Another novel feature of our study is the use of ICA, a powerful data-driven technique that utilizes higher order statistics to discover hidden factors underlying sets of random variables and signals to examine working memory abnormalities in schizophrenia. ICA is primarily a blind-source separation methodology and relies minimally on any *a priori* temporal information of the task itself [20]. This method is significantly different from a conventional fMRI analyses or seed voxel correlation analyses that rely upon strong assumptions of either spatial or temporal properties of the signal. ICA is a powerful tool to examine functional connectivity as the extracted signals for each component/network are by definition temporally correlated. The signals derived from ICA (component or network maps) represent functionally-integrated neural networks with unique profiles of blood oxygen level dependent (BOLD) response signal change across the fMRI timeseries. Therefore, the term “network” mentioned here refers to specific handfuls brain regions that share a similar timecourse as identified by ICA.

A modified fMRI version of the Sternberg item recognition paradigm [10], [29] was used to examine working memory functional connectivity differences between schizophrenia patients and healthy controls and to further characterize specific connectivity abnormalities during the distinct encoding and recognition phases of the experiment. We expected integrated functional networks to comprise the brain regions that we found active in our previous fMRI report using this task [10] – dorso/ventrolateral PFC, hippocampus, inferior/superior parietal lobule and anterior cingulate. We further hypothesized that we would find abnormal connectivity WM-engaged networks in schizophrenic participants, localized particularly to dorso/ventrolateral PFC and parietal regions. A secondary hypothesis was that increasing task difficulty (i.e., number of stimuli to encode) would alter both hemodynamic response amplitude [10], [23] and inter-regional functional connectivity in both groups. According to our theory of schizophrenia disconnection, such parametric task-difficulty effects should also reveal additional, specific schizophrenia related connectivity deficits. We predicted that schizophrenia patients would more greatly engage atypical brain regions to mediate higher task loads. A final aim of our study was to identify networks possibly contributing to schizophrenia abnormalities differences during probe recognition, which has been relatively understudied. Specifically, because we used a probe recognition type paradigm which is known to be somewhat easier than tasks with “free recall” demands, we expected that group differences in functional connectivity for the recognition phase of the task would be minimal. However, because such differences could still indicate pathophsyiology important to schizophrenia, we planned analyses to examine abnormality in recognition networks as well.

## Methods

### Participants

We examined 37 subjects with schizophrenia (mean age ± SD: 37.02±10.6 yrs; M∶F ratio 25∶12) who were participants in an ongoing study of psychosis at the Institute of Living and 54 healthy controls (33.2±11yrs; M∶F 27∶27) recruited from the community. Groups were matched demographically on age (*p* = 0.1) and gender (*Χ* ^2^ = 3.3; p = 0.08). Table 1 details the above demographic characteristics of both groups. All participants were assessed for DSM-IV Axis I disorder using the SCID-IV [30]. Exclusion criteria for healthy controls included any present or past Axis I disorder or family history of psychotic disorder and for all participants, any significant history of medical or neurological, head injury, or substance abuse within 6 months prior to participation. Fifteen schizophrenia patients were on antidepressants (trazodone (2), sertraline (2), escitalopram (3), fluoxetine (4), bupropion (2), venlafaxine (2)), seven were on mood stabilizers (divalproex (5), oxcarbazepine (1), gabapentin (1)), seven treated with first generation antipsychotics (chlorpromazine (1), haloperidol (2), fluphenazine (1), perphenazine (3)) and twenty two on second generation antipsychotics (risperidone (5), olanzapine (2), aripiprazde (4), quetiapine (6), clozapine (1), ziprasidone (5)). Three patients were on no antipsychotics. Medication information was unavailable for eleven patients who participated in the study. Participants gave written informed consent using procedures approved by the Yale and Harford Hospital institutional review boards.

**Table 1:** Demographic and behavioral characteristics of healthy controls and schizophrenia patients.

Subject Characteristics	Healthy Controls (n = 57) Mean+ SD	Schizophrenia patients (n = 37) Mean+ SD	T score	p value
Age	33.20+11.00	37.02+10.6	−1.54	0.12
Performance Accuracy (%)	0.95+0.05	0.82+0.12	7.27	<0.0001
			Chi-square	p value
Gender (M∶F)	27∶27	25∶12	3.30	0.08

### fMRI Task

Our task was a modified Sternberg Item Recognition Paradigm that required subjects to memorize a list of alphabetic letters (consonants only), maintain them in memory for several seconds, and then recognize whether probe letters were members of this list. During each encoding phase, subjects saw a list of consonants, displayed sequentially for 1.5 sec each with a 1 sec interstimulus interval (ISI). After a 9-sec maintenance period (during which they were asked to silently rehearse the consonant set presented), in the retrieval phase, subjects saw a sequential series of probe letters (onscreen for 2.5 sec with a 500 msec ISI) and were instructed to press one button with their dominant-hand index fingers for letters in the list (targets) and another button with the middle finger of the same hand for other letters (foils). An additional practice condition contained blocks of all possible memory loads. Each task condition lasted approximately 7 min. The task was implemented on standard desktop PCs running custom presentation software (VAPP, http://nilab.psychiatry.ubc.ca/vapp).

Before entering the scanner, all subjects were given complete task instructions and the practice condition. Practice and instructions were repeated if necessary until subjects achieved a high rate of correct responses. In the scanner, stimulus display was achieved with a rear-projection screen and a mirror mounted on the head coil; subjects made their responses with a fiber-optic response box (Photon Control, Burnaby, Canada).

Table 2 details the distribution of memory loads in the various task conditions. The above load size used in this study is optimal to investigate group differences as it aims to prevent floor ceiling effects at easier loads or heavier loads in patients or controls.

**Table 2:** Distribution of working memory loads in task conditions.

Load (Letters in Memory Set)	Total Probes*	Targets per Probe Set	Occurrences of Load per Condition
4	4	2	3
5	4	2	4
6	5	2 or 3	3

*The number of probes was varied across WM loads to achieve rough equality between the number of functional images acquired during the encoding and recognition. This was aimed to gain equal power to detection activation in both epochs with a fairly limited number of trials per subject.

### Data Acquisition

Functional MR images were collected at the Olin Neuropsychiatry Research Center in the Institute of Living/Hartford Hospital, using a Siemens Allegra 3T scanner (Siemens, Erlangen, Germany). A custom head cushion was used for head stabilization. T2*-weighted images were acquired with a gradient-echo planar sequence (TR = 1.86s, TE = 27ms, flip = 70°). The images consisted of whole-brain volumes of 36 sequentially acquired 4 mm slices parallel to the anterior commissure-posterior commissure line (voxel size 3.44×3.44×4 mm with a 1 mm slice gap). Behavioral data were acquired using the visual and audio presentation package (http://nrc-iol.org/vapp/).

### Data Analysis

Functional images were preprocessed with SPM2 (http://www.fil.ion.ucl.ac.uk/spm/software/spm2/). The first five images of each time series were removed to avoid T_1_ saturation effects. Data were realigned using the INRIAlign [31] toolbox. Motion parameters were visually inspected to only include data with translation motion less than 3 mm and rotational movement less than 1.5 degree in any direction. Realigned images were then spatially normalized to MNI standardized space using the EPI template image and spatially smoothed with a 12 mm isotropic kernel.

Time series were analyzed using a group ICA algorithm (GIFT v1.3c; http://icatb.sourceforge.com) [20], [32] to identify spatially independent and temporally coherent networks. The approach involved a standard method of pooling data from all participants into a single ICA analysis. Following this data reduction was carried out through two principal component analysis (PCA) stages which enables analysis of large data sets [20]. Data from all subjects was used to decompose and estimate twenty one mutually independent components using the Infomax approach [33]. The number of components was determined using the minimum length description (MDL) criteria adjusted to account for correlated samples [34]. This method produces for each component a spatial map (representing which brain regions comprise the network) and a timecourse of the BOLD signal change across the timecourse based on the overall group characteristics. Time courses and spatial maps were then back reconstructed for each participant. This back-reconstruction for each participant produced a series of spatial maps and component timecourses that captured individual differences in the expression of the ICA-derived component. This permitted standard random-effects hypothesis testing of group differences, condition differences, etc. Group analyses of spatial maps determined differences in degree of regional functional connectivity, while analyses of timecourse information allowed us to determine whether or not study groups differentially engaged the network during key phases of the fMRI working memory task. To identify and display the significant network brain regions, individual back-reconstructed subject components (pooled across patients and controls) were examined with random effects analysis (SPM2 one-sample *t* test) and overlaid on structural images (*p*<0.05 FWE, corrected for searching the whole brain). To examine network task engagement, component time courses were parameterized using multiple regression to provide association estimates (beta weights) between time courses and the phases of the fMRI paradigm (i.e., encoding vs. recognition). These beta weights represented the degree of synchrony between component time courses and the canonical hemodynamic response model, indicating whether or not the network represented in the component was engaged during that task phase. In addition to calculating beta weight coefficients for the overall encoding condition (collapsed over loads 4, 5 and 6), estimates were also derived for separate loads to investigate the effects of the same on encoding related neural systems. Initially, a one sample t-test was computed on the beta weights (pooled across both controls and schizophrenia from all components to find networks that significantly associated with each condition of the task. Next, *t* tests and correlation analyses were used to test study hypotheses.

Group differences in regional functional connectivity strength were identified through SPM2 random effects tests on the back-reconstructed spatial component maps. To test study hypotheses regarding differences in strength of regional functional connectivity between groups, a one-way analysis of variance was conducted on the association estimates to test for any significant group differences in the encoding and the recognition phases separately. For reporting purposes, images were converted from MNI to Talairach space using an available set of transformation scripts (http://imaging-mrc-cbu.cam.ac/uk/imaging/MniTalairach).

To test whether networks were differentially engaged during working memory task phases between the study groups, analysis of variance (ANOVA) compared the beta weights (dependent variable) between schizophrenia and controls (independent variable) in SPSS v15 (http://www.spss.com/spss/). To ensure that any group differences in network engagement were not unduly related to task performance issues, the results were confirmed using an analysis of covariance (ANCOVA) model controlling for task accuracy data. The above models tested whether the degree to which any component was engaged, differed between schizophrenia and control participants for either encoding or recognition. The above results were computed with a false discovery rate threshold that corrects for multiple comparisons.

The parametric nature of the Sternberg task also allowed us to interrogate task load dependencies in encoding-related networks for each group to determine the effect of memory load (4, 5, or 6 letters) on network response amplitude. To best depict meaningful group differences, we conducted a series of within-group SPM2 voxelwise correlation analyses separately for control and schizophrenia patients that assessed the association between individual back-constructed spatial component maps (representing regional strength of functional connectivity) and beta weights (representing the degree of encoding load-related task association). This analysis identified the subset of brain regions observed in the component spatial map that were more associated with the overall component timecourse when that timecourse more closely matched the canonical model of hemodynamic response expected during encoding at different loads. In other words, this supplemental analysis enabled us to identify and visualize which brain regions became more functionally connected in the presence of greater working memory loads for each group.

Additionally we also performed a 2×2 repeated measures analysis of variance on the association estimates to test for any significant interactions between condition (encoding, recognition) and diagnosis group.

Working memory task performance for each participant was assessed by computing the average percentage of correct responses during fMRI scans. A significant group difference in performance accuracy was determined using a two-sample *t* test. After the analyses described above identified which ICA components significantly discriminated the study groups, supplemental Pearson correlation analyses explored possible relationships between performance accuracy scores and ICA component beta weights (using the entire range of data across both groups). These latter analyses identified whether network engagement was related to overall task performance.

## Results

A systematic process was used to inspect and select the components of interest from the 21 estimated components. First, the association of each component's spatial map with *a priori* probabilistic maps of gray matter, white matter, and cerebral spinal fluid within standardized brain space (MNI templates provided in SPM2) helped to identify those components whose patterns of correlated signal change were largely consisted of gray matter versus non-gray matter. Components with high correlation to a priori localized CSF or white matter, or with low correlation to gray matter, suggested that they may be artifactual rather than representing hemodynamic change. As a result, five ICA components were discarded as representing signal artifacts (due to head motion, eye movement, ventricular pulsations, etc.) and 16 out of the 21 components were examined further.

As initially estimated using a one sample t-test, 13 of 16 components were associated with task activity during the encoding stage and 10 of 16 components correlated with the recognition phase of the task. However, as the main purpose of the paper was to investigate group differences in task associated network activity between controls and schizophrenia subjects, we do not elaborate on all components that were significantly associated with each task phase.

### Group Differences in Encoding Network Engagement

**Figure 1:**
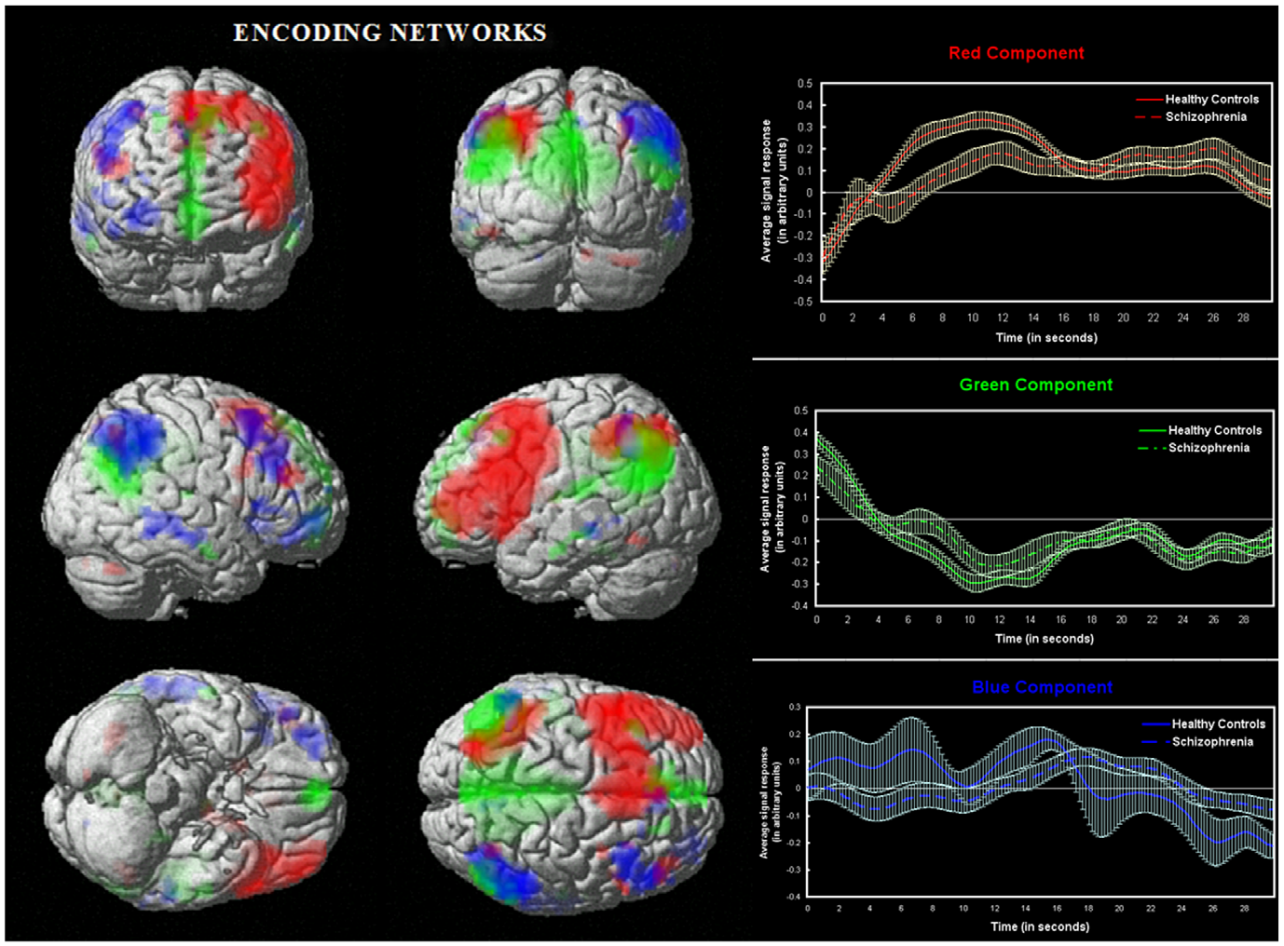
Spatial representation of networks that significantly differed between groups during encoding. 3D rendering of three distinct component networks that were significantly less engaged in schizophrenia during the encoding phase of the Sternberg working memory paradigm. The red network comprises of a highly left-lateralized network of DLPFC, VLPFC and Inferior/Superior Parietal regions. The blue network is comprised of a right-lateralized network of DLPFC, inferior frontal, inferior-superior parietal and middle temporal regions. The green network represents the “default mode” network representing the precuneus, anterior/posterior cingulate and the medial frontal gyri. All networks shown were derived by thresholding a random effects map (1-sample t-test): at P<0.05 FWE corrected. Accompanying the spatial maps are corresponding event averaged component responses over the encoding phase of the experiment.

Out of the above thirteen encoding related networks, ANOVA revealed three circuits to be significantly different between groups during the encoding stage of the experimental task (Figure 1). These included: 1) left dorso/ventro-lateral pre-frontal cortex, left superior/inferior parietal, cingulate, basal ganglia component (shown in Red; p = 0.004), 2) right dorsolateral PFC, right superior/inferior parietal, right middle temporal network (Blue; p = 0.023) and 3) a component resembling the “default mode” or resting state network that comprised of posterior cingulate, anterior cingulate, medial frontal gyrus, lateral temporal cortex and inferior parietal regions (Green; p = 0.007). When a supplemental ANCOVA examined group differences using task performance accuracy as a covariate, the results were slightly different; but all three of the networks described above remained significantly different between groups. The significance values were as follows: Red – p = 0.001; Blue – p = 0.05 and Green – p = 0.009. On applying a false discovery rate (FDR) correction for multiple comparisons to the supplemental ANCOVA results, only the red and green networks remained significantly different. Figure 1 shows the associated event-averaged time courses (across controls and schizophrenia) for the Red-, Blue- and Green-labeled networks. Significant regions encompassed by the above networks are listed in Table 3. The time courses of all the above networks were positively correlated with the task regressor representing the encoding phase of the experiment (except the default mode which was negatively correlated).

**Table 3:** Significant regions for the red, blue and green components (in figure 1) that were associated with working memory encoding along with their Talairach coordinates and suprathreshold volume in cm^3^.

Encoding							
Component	Network regions	Brodmann Area	Left Vol in CC	Right Vol in CC	Total Vol in CC	Left Max T (x, y, z):	Right Max T (x, y, z):
**Red Component**	Middle Frontal Gyrus	46, 8, 9, 6, 10, 11, 47	23.3	1.6	24.9	11.1(−48,28,26):	6.4(39,42,20):
	Inferior Frontal Gyrus	9, 44, 45, 46, 6, 10, 47, 13	17	0.5	17.5	11.1(−48,10,19):	5.5(56,16,27):
	Superior Frontal Gyrus	6, 8, 10	9.9	3.4	13.3	11.3(−6,11,52):	11.0(0,20,52):
	Inferior Parietal Lobule	40, 7, 39	7.7	0.4	8.1	11.0(−33,−53,44):	6.2(33,−59,47):
	Precentral Gyrus	9, 44, 6	6	0	6	10.4(−48,9,13):	NS
	Precuneus	7, 19, 39	3.9	0.1	4	10.5(−27,−65,42):	5.2(30,−62,34):
	Superior Parietal Lobule	7	3.2	0.3	3.5	10.7(−30,−65,45):	5.9(33,−62,47):
	Cingulate Gyrus	32, 9	1.4	1.2	2.6	7.5(−6,25,37):	7.7(9,20,40):
	Insula	13, 47	1.9	0	1.9	9.8(−45,9,13):	NS
	Superior/Middle Temporal Gyrus	22, 38, 37	0.8	0	0.8	7.5(−53,9,2):	NS
	Supramarginal Gyrus	40	0.6	0	0.6	7.8(−36,−48,36):	NS
	Lentiform Nucleus/Striatum	Putamen, Globus Pallidus & Caudate	0.5	0.1	0.6	5.8(−21,1,11):	5.7(18,9,8):
	Thalamus	Ventral Anterior & Lateral Nucleus, Medial Dorsal Nucleus	0.4	0	0.4	6.9(−15,−5,11):	NS
**Blue Component**	Inferior Parietal Lobule	40, 7, 39	1.8	7.5	9.3	7.5(−56,−51,38):	11.6(56,−42,44):
	Middle Frontal Gyrus	6, 8, 46, 10, 11, 9	0	7.6	7.6	NS	8.2(39,14,52):
	Middle Temporal Gyrus	21, 22	0.6	3	3.6	6.4(−59,−32,−6):	9.0(62,−41,−6):
	Supramarginal Gyrus	40	0.3	2.8	3.1	6.9(−56,−51,36):	11.1(56,−54,36):
	Inferior Frontal Gyrus	45, 47, 46	0	1.8	1.8	NS	7.8(53,18,5):
	Superior Frontal Gyrus	8, 10, 9	0.1	1.6	1.7	5.4(−3,29,51):	8.6(39,20,52):
	Superior Parietal Lobule	7	0	0.9	0.9	NS	7.7(36,−65,50):
	Angular Gyrus	39	0	0.5	0.5	5.5(−56,−56,36):	8.8(53,−56,36):
**Green Component**	Precuneus	31, 7, 23, 39, 19, 18	14.8	11	25.8	12.3(−9,−69,26):	10.7(3,−48,30):
	Cingulate Gyrus	31, 23, 24	8.3	8.9	17.2	12.5(−3,−42,27):	13.1(0,−42,27):
	Posterior Cingulate	23, 30, 29, 31	6.9	5.5	12.4	13.1(−3,−42,24):	13.9(3,−46,22):
	Middle Temporal Gyrus	39, 21, 19, 22	4.3	1.5	5.8	10.3(−56,−63,25):	6.9(42,−63,25):
	Superior Temporal Gyrus	39, 22, 13, 29, 41, 42	3.2	1.8	5	10.1(−39,−57,28):	6.3(53,−52,16):
	Medial Frontal Gyrus	10, 11, 8, 9, 6	3	1.9	4.9	9.9(−3,52,−10):	9.3(3,55,−8):
	Inferior Parietal Lobule	7, 40, 39	4.3	0.2	4.5	10.2(−39,−62,45):	5.8(48,−65,39):
	Angular Gyrus	39	2.3	1.2	3.5	11.5(−39,−54,30):	7.9(50,−68,31):
	Supramarginal Gyrus	39, 40	2.9	0.5	3.4	11.3(−39,−54,28):	6.6(56,−63,31):
	Cuneus	7, 18, 30, 19	1.5	1.1	2.6	10.9(−6,−68,31):	9.7(0,−68,31):
	Paracentral Lobule	31, 5	0.6	0.8	1.4	9.1(−3,−30,43):	9.0(0,−30,43):

### Group Differences in Regional Functional Connectivity during Encoding

Group differences in the comparison of study groups' spatial maps (*p*<0.05 FDR corrected) for component 1 (Red) were detected primarily in the left middle frontal gyrus (x, y, z: −45, 18, 36; *t* = 2.93), left inferior frontal gyrus (x, y, z: −43.3, 34.4, −4.8; *t* = 3.85) and left superior parietal (x, y, z: −30, −60, 54; *t* = 3.62). For component 2 (Blue), spatial differences were more subtle and found to be mainly in the right middle temporal gyrus (x, y, z: 63, −27, −12; *t* = 3.98) and inferior parietal lobe (x, y, z: 48, −54, 45; *t* = 2.71). Similarly within component 3 (Green), regions including the anterior cingulate (x, y, z: 3, 54, 3; *t* = 3.8), medial frontal gyrus (x, y, z: 6, 54, 3; *t* = 3.69) and precuneus/posterior cingulate (x, y, z: −3, −51, 30; *t* = 4.02) predominantly differed between groups. Compared to controls all the above network regions showed less strength of functional connectivity in the schizophrenia group.

### Recognition Phase Group Differences

**Figure 2:**
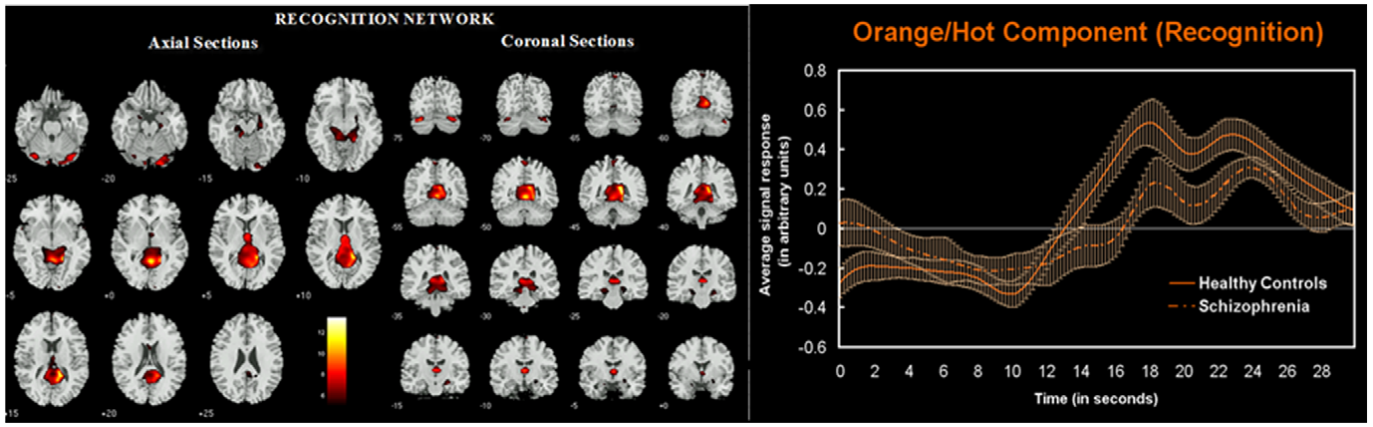
Spatial representation of the recognition network. Axial and coronal slices of network regions that behaved abnormally in schizophrenia during the probe recognition phase of the working memory task. Regions shown are thresholded at P<0.05 FWE corrected derived from a random effects analysis of the relevant component across all participants. Averaged fMRI response (with SEM): is shown on the right for the recognition phase of the task. Note the increased activity of this network during the recognition phase (albeit to a lesser extent in the schizophrenia group).

During the recognition phase of the experimental task one network was found to be engaged differently (p = 0.038) by the study groups: 4) posterior cingulate, cuneus, hippocampus/parahippocampus (shown in orange/hot in figure 2 along with event averaged hemodynamic responses for the two groups). However, upon accounting for task accuracy in an ANCOVA this network was no longer significant. Description of regions encompassed within this circuit is provided in Table 4. Spatial group differences in regional functional connectivity within the recognition circuit were noted in the left posterior cingulate (x, y, z: −3, −54, 21; *t* = 3.08) and bilateral cerebellum (x, y, z: −/+28, −72, −25; *t* = 3.03).

**Table 4:** Significant regions and their corresponding Talairach coordinates for the network (shown in figure 2) dysfunctional in schizophrenia during working memory probe recognition.

Recognition						
Network regions	Brodmann Area	Left Vol in CC	Right Vol in CC	Total Vol in CC	Left Max T (x, y, z):	Right Max T (x, y, z):
Posterior Cingulate	30, 23, 29, 31	3.6	4.6	8.2	8.1(−3,−51,19):	11.3(9,−49,19):
Parahippocampal Gyrus	19, 30, 28, Hippocampus, Amygdala, 27, 36, 37, 35, 34	2.1	4	6.1	6.9(−9,−46,5):	11.5(18,−47,−3):
Thalamus	Pulvinar, Medial Dorsal, Anterior & Ventral Lateral Nucleus	1.4	2.5	3.9	9.6(−6,−11,9):	9.8(9,−23,9):
Lingual Gyrus	19, 18, 17	0.9	2.1	3	8.1(−9,−85,−13):	11.1(18,−47,0):
Fusiform Gyrus	18, 19	0.2	1.2	1.4	7.6(−30,−74,−14):	9.2(24,−85,−16):
Inferior Occipital Gyrus	18, 17	0	0.7	0.7	NS	9.5(24,−88,−13):
Precuneus	7, 23	0.2	0.4	0.6	5.5(−3,−58,61):	6.6(3,−58,61):
Cuneus	30, 17	0.1	0.4	0.5	6.1(−6,−61,6):	10.0(9,−58,8):

### Working Memory Encoding Load Effects

**Figure 3:**
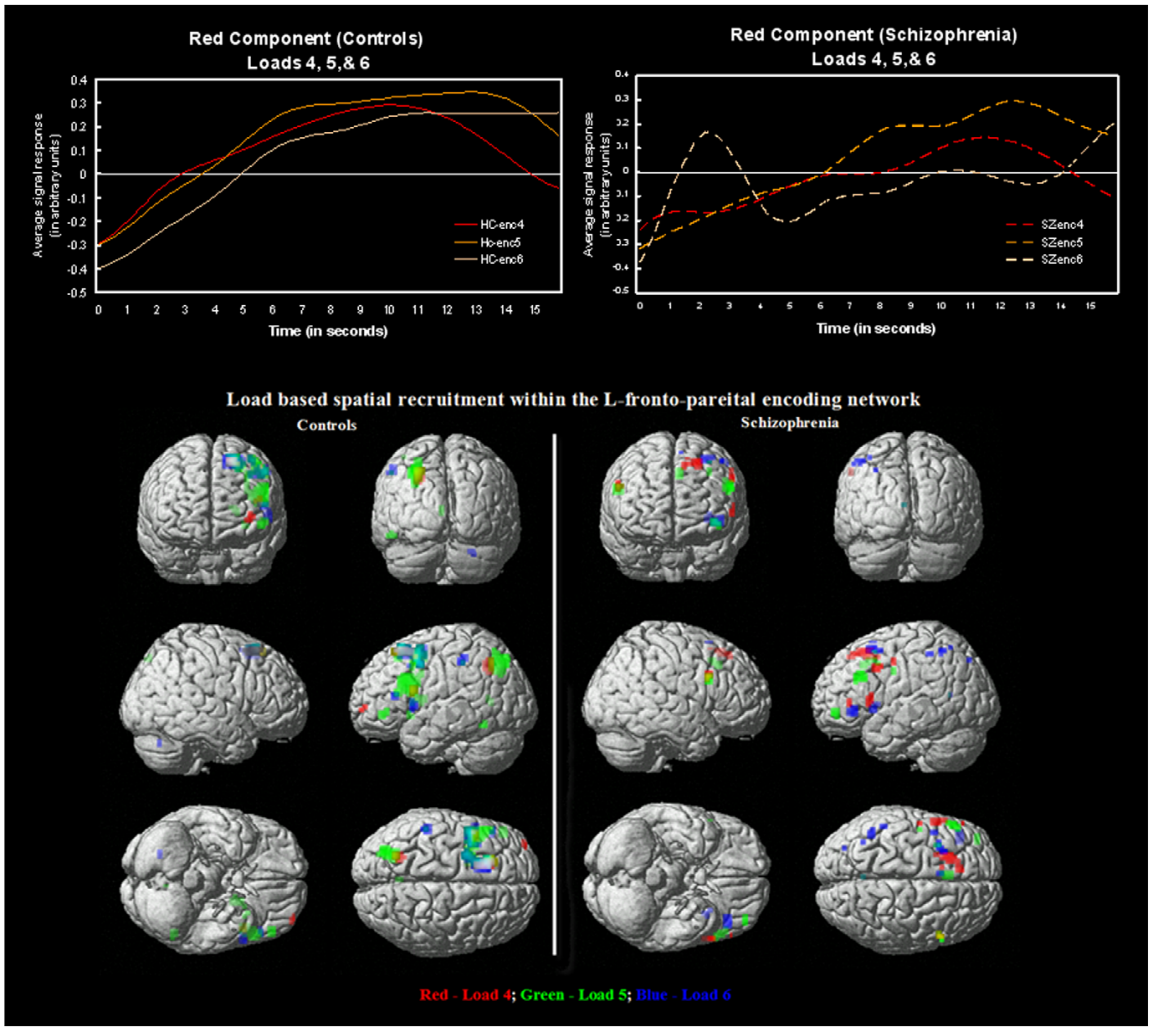
Functional recruitment of regions during different encoding loads. Event averaged time courses with standard error bars (top) and spatial regions correlated to beta estimates (bottom) for all three probe sizes (4, 5 and 6) for both the control and schizophrenia groups within the left-prefrontal-parietal encoding circuit. Regression results are thresholded at p<0.01 uncorrected level. Regions are color coded as follows. Red – load 4; Green – load 5 and Blue – load 6. Note, the absence of fMRI response in the posterior parietal and increased right PFC recruitment in the schizophrenia group (compared to controls): during lower loads suggesting a load-dependent functional disconnection of this neural network.

As shown in Table 5, the Red component was the only network that demonstrated significant (p<0.05 corrected or strong trends (p< = 0.07) in group differences across all three loads when examined separately. Hence the results of load effects presented in this paper primarily focus on this network. Figure 3 shows the event averaged time course across each load size (4, 5, 6) for both controls and schizophrenia patients for the fronto-parietal (Red) component 1. Figure 3 (top) shows that both controls and schizophrenic patients demonstrate a non-linear load dependent response of hemodynamic signal change for this neural system when encoding information. The fMRI response for load 4 was the lowest of all three loads, with response increasing and peaking the most with load 5 and dropping down again for load 6. This pattern was observed in both controls and schizophrenia patients. For all three loads, schizophrenia patients demonstrated lesser average amplitude of hemodynamic response than controls. Figure 3 (bottom) depicts SPM2 renderings of brain regions where regional strength of functional connectivity was associated with how strongly each spatial region within this network was engaged by encoding either 4, 5, or 6 stimuli (using the above described “third level” regression analysis). In general, both groups recruited ventrolateral PFC in addition to DLFPC and posterior parietal for higher cognitive loads. However, schizophrenia patients recruited right prefrontal areas more during lower loads than controls. In addition, we observed that the parietal regions were functionally disconnected in schizophrenia patients during lower loads (4 and 5) at the liberal statistical threshold examined (p<0.01 uncorrected). Table 5 shows corresponding mean beta weights for both groups and all 3 encoding and the single recognition network(s) across all loads.

**Table 5:** Mean/SD and group difference p values for beta estimates across all three loads and all four significant networks.

Components	Task Phase Associated	Enc 4 Beta Mean(SD)		Uncorrected p value without covarying for accuracy (HCvSZ)	FDR corrected p value covaried for accuracy (HC v SZ)	Enc 5 Beta Mean (SD):		Uncorrected p value without covarying for accuracy (HCvSZ)	FDR corrected p value covaried for accuracy (HC v SZ)	Enc 6 Beta Mean (SD)		Uncorrected p value without covarying for accuracy (HCvSZ)	FDR corrected p value covaried for accuracy (HC v SZ)	Recognition Beta Mean (SD)		Uncorrected p value without correcting for accuracy (HCvSZ)	FDR corrected p value covaried for accuracy (HC v SZ)
		HC	SZ			HC	SZ			HC	SZ			HC	SZ		
Red	***Encoding***	0.15 (0.24)	0.01 (0.36)	0.03	0.02	0.25 (0.22)	0.17 (0.31)	0.07	NS	0.25 (0.32)	0.05 (0.38)	0.009	0.03	0.001 (0.22)	0.07 (0.27)	NA	NA
Green	***Encoding***	−0.15 (0.3)	−0.01 (0.39)	0.07	NS	−0.26 (0.24)	−0.12 (0.27)	0.01	NS	−0.23 (0.36)	−0.11 (0.40)	NS	NS	−0.02 (0.20)	−0.07 (0.22)	NA	NA
Blue	***Encoding***	0.08 (0.36)	0.01 (0.27)	NS	NS	0.19 (0.38)	0.04 (0.17)	0.02	NS	0.09 (0.63)	0.05 (0.23):	NS	NS	0.24 (0.40)	0.19 (0.27)	NA	NA
Orange	***Recognition***	−0.29 (0.74)	−0.31 (0.55)	NA	NA	−0.23 (0.63)	−0.25 (0.50)	NA	NA	−0.27 (0.70)	−0.26 (0.60)	NA	NA	−0.007 (0.43)	−0.20 (0.35)	0.02	NS

### Group by Condition Interaction

Overall, analysis of beta weights found that two networks demonstrated a significant group (Controls vs. Schizophrenia) by condition (encoding vs. recognition) interaction. These were the 1) left dorso-/ventrolateral PFC-left posterior parietal-cingulate (Red) (p = 0.002) and the 3) default mode (Green) networks (p = 0.002). In schizophrenia, the former network demonstrated decreased engagement compared to controls during the encoding phase of the experiment and increased engagement during recognition. An opposite effect was observed in the latter network with schizophrenia participants showing increased functional synchrony during the encoding phase and vice versa for recognition relative to control participants.

### Behavioral Performance

Mean Sternberg task accuracy was significantly lower (*p*<0.0001) for the schizophrenia group (mean accuracy ± SD  = 0.82±0.12) compared to controls (0.95±0.05) assessed using a two-sample t-test. None of the subjects were performing near or below chance level (i.e. 50% accuracy). Further, Pearson correlations indicated that during encoding both the Red and Blue networks shown in Figure 1 correlated positively (*r* = 0.37; *p*<0.0001 and *r* = 0.20, *p*<0.05 respectively) with accuracy measures. The default mode/resting state network (Green) showed a negative association with accuracy (*r* = −0.30; *p*<0.004). The recognition-associated network did not significantly correlate with task accuracy.

## Discussion

The purpose of this study was to test for disconnection among prefrontal and parietal brain regions engaged for successful working memory performance in patients diagnosed with schizophrenia. We performed a single group ICA on all data followed by back-reconstruction to produce subject spatial maps and timecourses for each individual [20]. Performing a group ICA (i.e. collapsing over both groups) allowed us to identify functionally connected networks found in the entire sample, while preserving the individual participant/group changes [35]. Group ICA circumvents the problems such as noisy data, matching identical components across groups/subjects etc that are usually encountered by running separate ICA's. The initial idea of collapsing data over all loads was to investigate a main effect of task. In doing so, we were able to identify several task-related networks that were engaged to a lesser extent in schizophrenia patients, then extended these findings with supplemental analysis of load effects on functional connectivity for relevant networks.

### Red Network

The decreased functionality and anomalous behavior of the left fronto-cingulate-parietal-basal ganglia neurocognitive network observed in schizophrenia in our study is consistent with prior studies that have examined working memory-related BOLD activation [13], [21], [22], [36]. This network likely plays a crucial role in attention and executive control during working memory [37], [38]. Consistent with our results, previous studies have shown the importance of fronto-subcortical connections (that closely resemble the Red circuit from our study) during working memory in healthy adults and also have implicated abnormal connectivity of multiple regions within this network in various psychiatric disorders including schizophrenia [11], [12], [25]. In addition to group differences in hemodynamic response amplitude in this network, we also observed a slight lag in peak response for schizophrenia patients. This network also exhibited a load-dependent pattern of hemodynamic response amplitude; however, this dependency was non-linear in both the groups partly resembling an inverted “U” shape response in peakedness and latency. Overall, the network was less engaged in both spatial extent and amplitude in schizophrenia across all three task loads. A novel voxelwise regression analysis that analyzed spatial load patterns within this network captured individual spatial regions within this network that were associated or recruited during each load condition. This analysis suggested two striking dissimilarities between study groups. First, the increased recruitment of right prefrontal regions of during lower loads in schizophrenia raises the possibility that its elevated function represents a compensatory mechanism for deficits in their left prefrontal encoding network. Second, schizophrenia subjects failed to recruit parietal regions during lower loads. It is important to note that this novel analysis depicts differences in the degree of task modulation for each load-size condition rather than a strict measure of direct connectivity between regions as explored by previous studies. Together, these results illustrate differences in inter-regional connectivity and a distinct spatial pattern of network recruitment in schizophrenia within this key frontal-parietal working memory network, thereby providing further evidence for a “disconnection hypothesis” [18] of schizophrenia.

### Blue Network

We observed a right fronto-parietal circuit containing DLPFC that included portions of inferior frontal and temporal gyri that was associated with encoding and that engaged abnormally in schizophrenia. This suggests that this circuit might be related to deficits in encoding visuospatial stimuli during the task in schizophrenia that contribute to poor working memory performance. However, unlike the Red network (whose hemodynamic response peaks and tapers off during encoding) this network seems to be constantly engaged throughout the encoding period in both groups (albeit to a significantly lesser extent in schizophrenia). Importantly, the fact that these group differences in network dynamics were associated with task accuracy emphasizes the importance of these findings to neural network dysfunction in schizophrenia.

### Green Network

The third significant encoding-related network that was abnormal in schizophrenia consisted of brain regions including the anterior/posterior cingulate, medial frontal gyrus and inferior parietal regions. Together, these have been proposed to represent the default mode, or “idling state” of the brain. Previous studies have shown that these regions decrease activity with increasing cognitive load and might be involved with self-reflection processes, mental imagery and episodic memory retrieval [28], [39], [40], [41]. Consistent with prior studies [35], [41], [42], we found this circuit to act abnormally in schizophrenia in that it was significantly less engaged (negatively modulated) during encoding, had abnormal load-dependent modulation during both encoding and recognition, and was also negatively correlated to performance accuracy (for schizophrenic patients who performed more poorly overall, with lower accuracy scores).

### Orange Network (Probe Recognition)

During probe recognition we found abnormal engagement of a hippocampus, posterior cingulate, cuneus and cerebellum network. These regions previously have been implicated in contributing to working memory deficits in schizophrenia during verbal working memory recognition/retrieval [15], [17]. In addition, one previous study also found similar visual association areas to be impaired in early stages (encoding) of working memory in adolescents of schizophrenia [43]. This lends further support to our initial hypothesis of similar regions being affected during both encoding and recognition. Hemodynamic time course averages for this component suggest that it is evenly active almost throughout the recognition phase (again lesser amplitude and slightly lagged in peak response for schizophrenia). Because our task involved recognition of probe items rather than a more challenging free recall demand, it is reasonable to expect fewer executive networks to behave abnormally as the demands on cognitive processing during this phase are relatively low. However, given that this network was no longer significant when ANCOVA statistically adjusted for differences in performance accuracy, these results should be interpreted with caution.

This study in general provides further support for the “disconnection syndrome” hypothesis. The novelty of our study lies in the fact that we utilize ICA to identify which functional networks engage abnormally in schizophrenia specifically during working memory and to characterize various types abnormal activity dynamics in distinctly different networks which ultimately might point towards specific pathophysiological mechanisms. It is important to note that some of the networks described above are known to be engaged during several cognitive tasks and may not just be limited to the present working memory paradigm. For example, a number of these circuits are identified during performance of various tasks when analyzed using ICA [35], [39], [40], [44]. Others (e.g., the “default mode”) appear to be near-ubiquitously engaged across different task contexts). One potential limitation of our study was our inability to investigate the maintenance condition of the working memory task due to our fMRI task design. However, even though our task design did not allow us to do this we would like to emphasize that brain regions engaged for encoding information into working memory largely overlap with regions involved with active maintenance or manipulation of that information [1]. Another study limitation is that we were not able to disambiguate possible medication effects in patients (due in part to lack of complete pertinent data) that might have influenced schizophrenia network abnormalities. In addition, given the nature of the sample collected and their behavioral performance we are unable to address a perennial problem in fMRI research of schizophrenia which is how brain network differences attributed to disease can be disentangled from that due to poor performance. However, our supplemental performance-based ANCOVA results lend confidence in the robustness of the group differences detected.

In summary, we demonstrate a significant impairment of the engagement of a distributed working memory neural network comprising bilateral PFC, anterior cingulate, medial temporal, basal ganglia, inferior frontal and bilateral posterior parietal regions primarily occurring during stimulus encoding. We also provide support for the disconnection hypothesis in schizophrenia by showing that the left prefrontal-parietal network demonstrates an abnormal load dependent neural pattern both in terms of regional connectivity and hemodynamic response (i.e., different network dynamics). These results indicate that impaired working memory ability in schizophrenia is related to abnormal functional integration of several distinct, but potentially interacting networks of brain regions. While some of what is demonstrated here has been previously suspected or could be inferred from similar prior research, one clear value in the current study is precise delineation of specific of network connectivity disruption during working memory. This is an important step towards future studies that will focus further on further characterizing how these networks fail to engage, the clinical or cognitive significance of specific patterns of disrupted connectivity in various abnormal networks through associations with symptomatology or neurocogntive data, and assessment of the relationship between functional disconnection markers and schizophrenia risk genotypes.
